# Monitoring of Active Human Herpes Virus 6 Infection in Iranian Patients with Different Subtypes of Multiple Sclerosis

**DOI:** 10.1155/2013/194932

**Published:** 2013-01-22

**Authors:** Nourollah Ramroodi, Nima Sanadgol, Zohre Ganjali, Abbas Ali Niazi, Vida Sarabandi, Ali Moghtaderi

**Affiliations:** ^1^Department of Neurology, Zahedan University of Medical Science, Zahedan 43181-98168-43175, Iran; ^2^Department of Biology, Faculty of Science, Zabol University, Zabol 98613335856, Iran; ^3^Cellular and Molecular Research Center, Tehran University of Medical Sciences, Hemmat Campus, Tehran 1449614535, Iran; ^4^Department of Pathology, Zahedan University of Medical Sciences, Zahedan 43181-98168-43175, Iran

## Abstract

*Background*. Recently, it has been suggested that human herpes virus 6 (HHV6) may play a role in the pathogenesis of multiple sclerosis (MS). Our purpose is to determine the incidence of reactivated HHV6 in MS patients. *Methods*. Viral sequence analyzed by qPCR in the peripheral blood mononuclear cells (PBMCs), serum, and saliva samples of different subtypes of MS patients (*n* = 78) and healthy controls (*n* = 123). HHV6 IgG and IgM antibody levels measured by ELISA technique in the plasma samples of both groups. Likewise, cerebrospinal fluid (CSF) samples of some MS patients (*n* = 38) were analyzed for viral sequence. *Results*. Results demonstrate increased levels of anti-HHV6-IgG (78.2% versus 76.4% in controls; *P* = NS), and IgM (34.6% versus 6.5% in controls; *P* < 0.05) in MS patients. Furthermore, RRMS and SPMS patients showed relatively higher anti-HHV6 IgG and IgM compared to PPMS (*P* < 0.001). Moreover, load of cell-free viral DNA was higher in RRMS and SPMS patients and detected in 60.2% (47/78) of MS patients, compared with 14.6% (18/123) of healthy controls (*P* < 0.001). Moreover, load of cell-free viral DNA was higher in RRMS and SPMS patients and detected in 60.2% (47/78) of MS patients, compared with 14.6% (18/123) of healthy controls (*P* < 0.001). *Conclusions*. The results extend the observation of an increased frequency of systemic reactivated HHV6 infection in MS patients with developed stages of disease.

## 1. Introduction

Human herpes virus 6 (HHV-6) belongs to the beta-herpes virus subfamily of the Herpesviridae family, with a linear double-stranded DNA genome of 160 kb [1]. After primary infection, HHV-6 remains latent in lymphocytes and monocytes unless the immune system is compromised, whenever the virus reactivates [2–4]. Multiple sclerosis (MS) is the most prevalent demyelinating disease among young adults, affecting many people in developing countries [5]. The prevalence of MS, according to World Health Organization reports (2006), should be about 18 to 175 in 100,000, according to geographic distribution of the disease. However, a recent study has shown that Iran could be considered as an area with a medium to high risk of MS and in southeastern Iran the incidence rate is showing a faster growth rate, compared to previous years [6–8]. Relapsing Remitting MS (RRMS) is the most frequent (85%–90%) forms of MS and affects women about twice as often as men. Most RRMS patients develop Secondary Progressive MS (SPMS) later. About 10%–15% of patients present with insidious disease onset and steady progression, termed Primary Progressive MS (PPMS). It is not clear which factors are responsible for the different courses [9]. No virus has been definitively implicated as a causative factor of MS, but certain HHVs have been linked with the development of MS [10, 11]. Since the first papers linking HHV-6 to MS appeared in 1993 [12] many others have presented contradictory results on its possible role in the disease. Some authors defended the association [13–19], whereas others denied it [20, 21]. Anyway, human herpes virus 6 is a very probable candidate in MS because it is neurotropic and a primary infection with this agent may cause several neurologic complications; it is characterized by latency and periodic reactivation; and it is ubiquitous [22–25]. A number of hypotheses have been proposed to explain how HHV-6 may act as a causative agent in MS, including direct cytopathic action, molecular mimicry or modulation of cytokine production during acute infection or virus reactivation, and an increase in an already present immune response during virus reactivation, a phenomenon also known as bystander effect [26–28]. The combination of all of these studies enhances the viral hypothesis and makes HHV-6 a credible virus for involvement in multiple sclerosis. In this case-control study we attempted to focus in both HHV-6 antibody and genome studies to flawless appraise the systemic reactive HHV-6 infection in Iranian MS patients. In this research for the first time, we used wide spectrum of clinical materials and methods together to evaluation role of HHV-6 reactivation in development of different MS courses. 

## 2. Material and Methods

### 2.1. Patients and Samples

The study, approved by the Zahedan University of Medical Science Multiple Institutional Review Board, was conducted with all clinical samples from MS patients who were treated at the Department of Neurology, Ali-ebn Abitaleb Hospital, Zahedan, Iran, and Healthy Blood Donors (HBD) who voluntary submitted for research at the central medical laboratory of Zahedan from December 2008 to July 2009. MS patients (in southeast of Iran) who had been diagnosed with Magnetic Resonance Imaging (MRI) and McDonald criteria were collected [29]. We analyzed 201 different samples; 78 patients and 123 people as the healthy control group. The patient group comprised 22 men (mean age, 28.8 years; age range, 17–48 years) and 56 women (mean age, 30.3 years; age range, 16–52 years). The control group of healthy blood donors comprised 34 men (mean age, 26.4 years; age range, 17–42 years) and 89 women (mean age, 26.0 years; age range, 17–50 years). The Expanded Disability Status Scale (EDSS) score for all patients at the inclusion time were below scale 5.0, except of 3 individuals with SPMS (scale 6.5) and 5 with RRMS (scale 5.0). All patients had at least annual relapse rate 1, during 2 years before inclusion in the study. Serum, plasma, PBMCs and unstimulated whole saliva samples were collected by standard methods described previously [30]. A total of 23 CSF samples (1.5 mL) were also collected from MS patients (RRMS = 22, SPMS = 6, PPMS = 10) from a Lumbar Puncture (LP) in to sterile tubes and centrifuged for 15 min at 180 g at 20°C to obtain cell-free supernatants. Samples (Serum, plasma, PBMCs, saliva and CSF) from 11 patients with RRMS and 6 patients with SPMS (17 samples in total) were obtained during periods of disease exacerbation and the relation was tested between defined HHV-6 reactivation periods and exacerbation rate for a mean of 1 year. Patients did not receive any kind of drug treatment at least 1 week prior to sampling. All Specimens were stored at −70°C until the experiment was performed. When multiple specimens were submitted for one patient, all of them were tested more than once and mean of them used for analysis.

### 2.2. DNA Extraction and Quantitative Real-Time PCR

HHV-6 DNA extraction was performed on 100 *μ*L of samples using RIBO-prep nucleic acid extraction kit (Interlabservice, Moscow, Russia) according to the manufacturer's protocol. Real-time PCR was performed using the AmpliSens HHV6-screen-FRT kit (Interlabservice) according to the manufacturer's protocol. This real-time PCR assay was shown to be sensitive, specific, and reproducible (*Sensitivity*: 400 copies/mL or 5 DNA copies per 10^5^ cells). The assay detects both subtypes 6A and 6B, and the primers and probe do not cross-react with the specificity panel selected for the assay. The assay has an internal control, which allows inefficient extraction or PCR inhibition to be detected. Real-time amplification was carried out using 10 *μ*L DNA eluate combined with 10 *μ*L PCR-mix-1-FL and 5 *μ*L PCR-mix-2-FL using Rotor-Gene 3000 Instrument (Corbett Research, Sydney, Australia) with the following cycling parameters: pre-denaturation at 95°C for 15 min, 95°C for 5 s, 60°C for 20 s and 72°C for 15 s for 45 cycles. Data acquisition was performed in both Cy5/Red channel for HHV-6 DNA and in the FAM/Green channel for *β*-Globin gene DNA during the annealing (60°C) stage. For quantification of HHV-6 DNA two standard positive sample KSG1 (10^4^ copies per reaction mixture) and KSG2 (10^2^ copies per reaction mixture) were included in the run (Interlabservice). Calculations of C_*t*_, preparation of standard curve and quantification of DNA in each sample were performed by Rotor-Gene Operating Software, version 1.8 (Corbett Research). 

### 2.3. HHV-6 Antibody Responses

Concentrations of plasma anti-HHV6, IgG, and IgM were measured based on Enzyme-Link Immunosorbant Assay (ELISA) in an automated instrument, according to the manufacturer's instructions (PANBIO, Windsor, Australia). Briefly, 100 *μ*L of sera diluted 1 : 100 were added to the wells coated with the HHV-6 viral lysate for anti-HHV-6 assays. Samples were incubated 20 to 60 minutes at 37°C and washed five times. One hundred microliters of horseradish peroxidase conjugated antihuman IgM or IgG was added to each well and incubated 20 minutes at 37°C. After washing five times, 100 *μ*L of TMB was added, incubated 10 to 20 minutes at room temperature, and reaction was stopped and read in an ELISA reader at 450 nm. Each plate contained positive, negative, and cut-off control sera. In addition, the assays were validated in our laboratory by using sera from HHV-6 PCR-confirmed infected individuals. Results were expressed using arbitrary units (ELISA titers). Anti-HHV-6 assays were expressed using PanBio units = 10 × absorbance of sample/mean absorbance of cutoff. PanBio units > 20 were considered positive for IgM and PanBio units > 11 were considered positive for IgG. 

### 2.4. Viral Reactivation Markers

In this study, we considered reactive HHV-6 infection, when detected two positive (for both IgG and IgM) by immunoassay and/or two or more consecutive positive qPCR and/or load HHV-6 ≥ 200 copies in serum or ≥ 150 copies in PBMCs. 

### 2.5. Statistical Considerations

Statistical analysis was performed using Fisher test comparing the incidence of HHV6 in controls and MS patients. The *χ* ^2^ test was used to analyze the significance of differences in serology and DNA detection. All *P* values are Two-tailed and significant at *P* < 0.05 or *P* < 0.01 depending on statistical method.

### 2.6. Ethical Considerations

The study conformed to the Helsinki Declaration and was reviewed and approved by the local research committee; written informed consent was obtained from all subjects.

## 3. Results

### 3.1. Detection of IgG and IgM Antibodies against HHV-6

Recent studies have demonstrated that at least 78.2% of MS patients are positive for HHV-6 specific IgG (IgG^+^) antibodies in contrast with 76.4% of healthy controls (Table 1). 100% of SPMS patients were IgG^+^ in their serum samples compared to 82.6% of the RRMS, and 57.1% of PPMS samples (Table 2). The frequency of HHV-6 specific IgM (measuring reactive infection) in normal population was 6.5% compare with 34.6% of MS patients (Table 1). 36.3% of SPMS patients were IgM^+^ in their serum samples compared to 47.8% of the RRMS, and 4.7% of PPMS samples (Table 2).

### 3.2. Load of HHV-6 Genome in Clinical Samples

HHV-6 DNA was detected in serum of 60.2% (47/78) of MS patients and only 14.6% (18/123) of healthy controls (Table 1). As shown in Table 2, 76.0% (35/46) of patients with RRMS, 63.6% (7/11) of patients with SRMS and 23.8% (5/21) of patients with PPMS had HHV-6 DNA in their serum. HHV-6 DNA was detected in PBMCs of 66.6% (52/78) of MS patients and with evidence of latent HHV-6 infection and only 41.4% (51/123) of healthy controls (Table 1). 78.2% (36/46) of patients with RRMS, 63.6% (7/11) of patients with SRMS and 42.8% (9/21) of patients with PPMS had HHV-6 DNA in their PBMCs (Table 2). As with the saliva samples, 11.5% (9/78) of the patients had viral DNA compared to 2.4% (3/123) of the controls (Table 1). HHV-6 DNA was detected only in ten CSF samples of RRMS (21.7%) and one CSF sample of SPMS (9.0%) during an exacerbation (relapse) but was not found in CSF of patients with remission or patients with PPMS (Table 2). Viral DNA was found in all saliva samples that were previously positive for viral DNA in their PBMCs both in patients and controls. No amplifiable viral sequence was found in CSF of PPMS patients, and PBMCs showed higher prevalence of viral sequence compared to saliva samples in both patients and controls (*P* > 0.005).

### 3.3. Systemic HHV-6 Infection and Disease Exacerbation

Reactive viral infection in these patients was confirmed by the detection of specific anti-HHV-6 IgM antibodies in their plasma (Table 2). As a measure of reactivation, combined qPCR results and IgM serology showed 32.0% (25/78) of the patients had reactive HHV-6 infections, in contrast to none of the controls (Table 1). Viral DNA in serum and specific IgM antibodies in plasma were not detected in 88.6% (109/123) of healthy controls. Ten patients with RRMS and only one patient with SPMS showed the further positivity in all specimens (Table 2). The risk of an exacerbation of MS calculated according to the type of HHV-6 infection (active or latent) was 4 times higher for the patients with HHV-6 reactivation (*P* > 0.005). We found a positive correlation between the detectability of HHV6-DNA in CFS from patients undergoing exacerbation and also decrease in HHV6-IgG/IgM ration in this group. Episodes of defined HHV-6 reactivation were observed in a subgroup (8 patients with RRMS and 6 patients with SRMS), and these episodes were associated with increased risk ration (RR) for disease exacerbation. In these subgroup patients, the annual number of reactivation was 3.10 in the group of 8 patients who had one or more relapses, compared with 1.12 in the group of 6 patients who did not experience a relapse (*P* < 0.05). In a 4-week period beginning 2 week before the reactivation and ending 2 weeks after the reactivation, the relative risk of relapse was 3.5 (*P* < 0.05) compared with all other periods. 

### 3.4. Correlations between Seroanalysis, HHV-6-DNA Detection, and Gender

**Figure 1:**
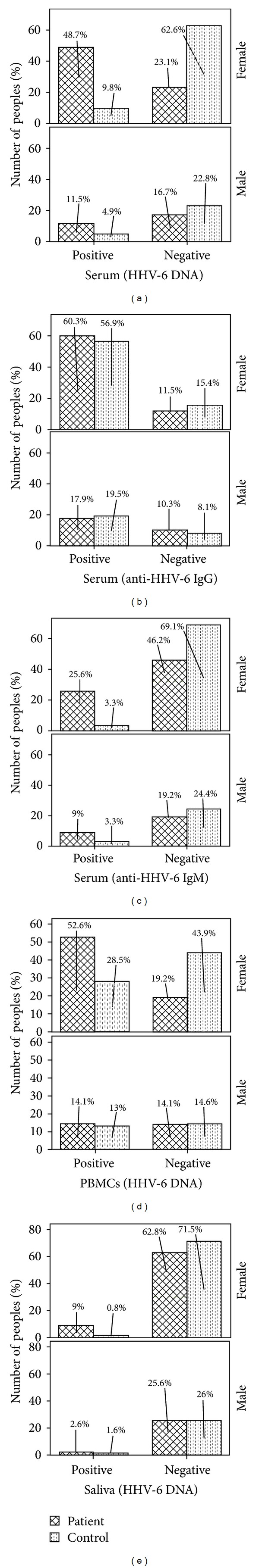
Prevalence of HHV-6-DNA and its antibodies among male and female in healthy controls and MS patients.

**Figure 2:**
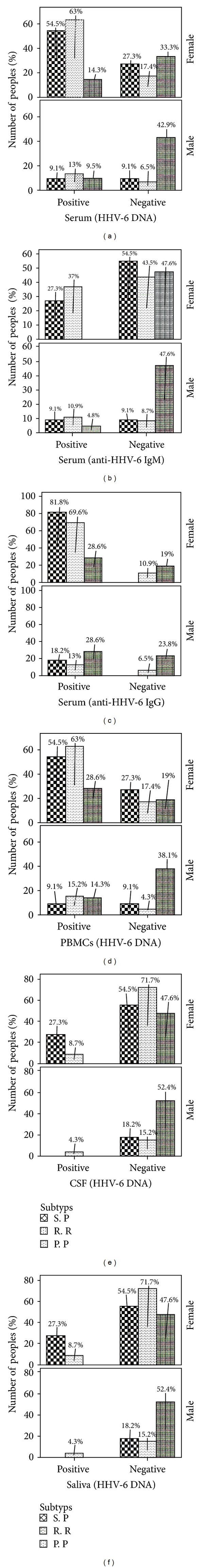
Prevalence of HHV-6-DNA and antibodies among male and female in different subtypes of MS.

Significant correlation between viral sequence detection in specimens and an increase in antibody response was not observed in patients (Table 3). Neither viral DNA in serum nor the presence of IgM specific antibodies or elevated titers of IgG antibodies to HHV-6 was found in 8.6% (4/46) of RRMS, 18.1% (2/11) of SPMM and 40.9% (9/22) of PPMS, confirming that in these patients HHV-6 infection remained latent. Significant difference and positive correlation with concentration of HHV-6-DNA and HHV-6-IgG in plasma was found only in control group (*P* < 0.01), but a significant inverse correlation with HHV-6-DNA in saliva and IgM response were found for both groups (Tables 3 and 4). Correlation was found between detection of HHV6-DNA in serum and detection of HHV-6-DNA in CSF (*P* < 0.05) and PBMCs (*P* < 0.05) of patients (Table 3). Serologically, immune status showed poor correlation with IgM concentration and detection of HHV6-DNA in serum of patients (Table 3). There were no statistically significant correlations between detection of HHV6-DNA in serum and HHV6-DNA in PBMCs in controls (Table 4). Data showed direct correlation between HHV-6-IgM concentration and detection of HHV6-DNA in serum and IgG response in patients (Table 4). Again, a positive correlation was observed between increase HHV6-IgG concentration and HHV6-DNA in saliva and PBMCs only in controls (Table 3). For demonstrate prevalence HHV-6-DNA and anti-HHV-6 antibodies, comprehensive analysis performed among males and females in both control and patient groups (Figures 1 and 2). In all cases, female patients showed more positivity (Figure 1) and systemic HHV-6 infection were found more in females compared with males (*P* < 0.001). Female patients with RRMS showed higher prevalence in HHV-6-DNA (serum and PBMCs samples) and had high titer of anti-HHV-6 IgM compare with both males and other subtypes (Figure 2). Increased HHV-6-DNA concentrations tended to be associated with HHV-6 systemic infection, but associations with additional components such as MS subtypes and gender were even stronger.

## 4. Discussion

A viral trigger involved in multiple sclerosis has been suggested more than 100 years ago [31], and an extensive list of candidate viruses has emerged since then. The frequency of HHV-6 specific IgG (measuring latent infection) in normal population was 76.4%, relatively consistent with the average global frequency of 90% [32]. Several clinical studies have suggested that MS in general as well as episodes of disease exacerbation are associated with concomitant viral or microbial infections [33–35]. Viruses may play a role, since MS relapses are often associated with common virus infections [36]. HHV-6 may directly lyses and thus destroys infected target cells or it may induce inflammatory and autoimmune reactions. These can be mediated by a large variety of HHV-6-induced or altered cytokine and chemokine patterns as well as by modulation of cell membrane receptors [37]. MS is usually diagnosed in the second or third decade of life, and it is difficult to prove a causative association with HHV-6 infection, which in the event of acute infection during childhood does not usually produce acute after-effects. In most studies, HHV-6 has been found in normal control samples and is frequently absent in some of the multiple sclerosis samples [38, 39]. This is especially apparent in the majority of studies that only examine sera and/or CSF or cell free DNA [40–44]. HHV-6 reactivation has been documented in small subsets of patients in several diseases [45]. As noted earlier, most cases of HHV-6 reactivation are benign; even though the virus is present and replicating, patients remain asymptomatic in most cases. Very little is known about the prevalence of HHV-6 in Iranian MS patients or in the general population of the country. HHV-6 is widespread throughout the world, with geographic differences in HHV-6 prevalence varying between 70 and 100% [46, 47]. This study supports the role of HHV-6 in the pathogenesis of MS by suggesting that the presence of systemic HHV-6 infection coincides with clinical worsening in a subset of patients. Analysis of serum HHV-6 DNA demonstrated that there is a statistically greater likelihood of detecting HHV-6 DNA in the CSF of a RRMS patient than other courses. We hypothesized that there may be multiple “triggers” by which foreign antigens, including infectious agents, may be associated with immune attacks on the CNS. We propose that HHV-6 may be one such trigger and if so, the mechanism(s) by which this virus is associated with the pathogenesis of MS would be important to define. HHV-6 DNA detection in PBMC and salivary glands has no clinical relevance because the virus can be latent in them and its presence does not discriminate between active infection and latent stages [48, 49]. RRMS patients had significantly higher prevalence of plasma HHV-6 IgM than other patients. Increased IgM antibodies to HHV-6 could represent an immune response associated with a more recent exposure to this virus and would be consistent with the hypothesis that this virus may be linked with MS pathogenesis. Our results are in accordance with studies that reported a higher PCR positivity for HHV-6 in serum and CSF of patient with developed stages (RRMS and SPMS) and also higher concentration of HHV-6 IgM in patients with exacerbation [50–53]. High levels of HHV-6 DNA have been detected in the serum and CSF of MS patients especially in relapse course in contrast with other courses and controls. MS patients have increased titers of plasma antibodies reactive with HHV-6, and 34.6% of them are positive for HHV-6-IgM antibodies. In spite of high prevalence of latently infected individuals in the healthy population, present of high reactivation of HHV-6 in patients with RRMS establish a causative role for HHV-6 in exacerbation of MS. Recently, it has been shown that 52.5% of peripheral blood mononuclear cells from MS patients harbor HHV-6 DNA are in a latent, nonproductive form, similar to the case for the control population. Therefore, to establish a correlation, it is necessary to discriminate between latent and productive infections. The association of HHV-6 with obtaining to MS remains mysterious and a more extensive understanding of HHV-6 neurotropism and its association with the disease process is required.

## 5. Conclusions

The reactivation of HHV-6 infection in MS patients was supported by serological investigations and molecular detection. As prevalence of anti-HHV-6-IgG in plasma and HHV-6-DNA in PBMCs was equivalent in both experimental groups, we assumed that both patients and controls have previously had an active infection and then establish a latent infection. Alternatively, because of high copy number of HHV-6 DNA in serum and also lower titer of anti-HHV-6-IgG in contrast with anti-HHV-6-IgM observed in patients with RRMS, we proposed that reactivation could have occurred in this group. On the other hand, the presence of HHV-6 DNA in CSF samples, which is a reliable indicator of reactive viral infection, was detected only in patients with RRMS, and strongly validated our hypothesis. The absence of HHV-6-DNA in CSF of some patients with active MS may be associated with an early stage of viral replication. Although this study is prospective in design, we cannot definitively prove that HHV-6 plays a causative role in MS. We emphasize that only through well-controlled interventional clinical trials with effective and safe antivirals can a causal role be made for any infectious agent in MS.

## Figures and Tables

**Table 1:** Prevalence of HHV-6-DNA (copies/mL) and HHV-6-antibodies (U/mL) among controls and MS patients. HHV-6-DNA was analyzed in serum via qPCR as described previously. Concentration of plasma anti-HHV-6, IgG and IgM were measurement in an automated instrument, according to the manufacturer's instructions. Data are representative of three independent experiments.

	Patients (*n* = 78)	Controls (*n* = 123)	
	P (%)		Sig. (2-tailed**)**
	[mean ± SD]		
Anti-IgG (U/mL)	61 (78.20) [15.54 ± 1.90]	94 (76.42) [12.37 ± 1.59]	*P* = NS
Anti-IgM (U/mL)	27 (34.61) [24.90 ± 1.85]	8 (6.50) [26.97 ± 2.10]	*P* < 0.05
Saliva-DNA (copies/mL)	9 (11.53) [127 ± 11.00]	3 (2.43) [152 ± 18.33]	*P* < 0.05
Serum-DNA (copies/mL)	47 (60.25) [264 ± 51.51]	18 (14.63) [192 ± 54.19]	*P* < 0.001
PBMCs-DNA (copies/mL)	52 (66.66) [165 ± 41.38]	51 (41.46) [157 ± 32.57]	*P* < 0.05

PBMCs: peripheral blood mononuclear cells; CSF: cerebrospinal fluid; P: positive; NS: not significant.

**Table 2:** Prevalence of HHV-6-DNA (copies/mL) and HHV-6-antibodies (U/mL) among different subtypes of MS. HHV-6-DNA was analyzed in serum via qPCR as described previously. Concentration of plasma anti-HHV-6, IgG and IgM were measurement in an automated instrument, according to the manufacturer's instructions. Data are representative of three independent experiments.

	Saliva	Serum	PBMCs	CSF	Anti-IgG	Anti-IgM
			P (%)			
			[mean ± SD]			
MS (*n* = 78)						
(1) RRMS (*n* = 46) CSF (*n* = 22)	6 (13.04) [127 ± 12.43]	35 (76.08) [272 ± 39.15]	36 (78.26) [178 ± 42.60]	10 (45.45) [128 ± 7.58]	38 (82.60) [15.96 ± 1.43]	22 (47.82) [24.85 ± 1.92]
(2) SPMS (*n* = 11) CSF (*n* = 6)	3 (27.27) [128 ± 9.84]	7 (63.63) [294 ± 44.77]	7 (63.63) [151 ± 14.44]	1 (16.66) [145]	11 (100) [16.51 ± 2.27]	4 (36.36) [25.67 ± 1.43]
(3) PPMS (*n* = 21) CSF (*n* = 10)	0 (—) [—]	5 (23.80) [165 ± 12.45]	9 (42.85) [125 ± 7.23]	0 (—) [—]	12 (57.14) [13.32 ± 1.17]	1 (4.76) [23.05]

Sig. (2-tailed)						
Subtypes (1), (2)	NS	NS	P < 0.05	NS	NS	NS
Subtypes (1), (3)	—	P < 0.001	P < 0.001	—	P < 0.001	NS
Subtypes (2), (3)	—	P < 0.001	P < 0.001	—	P < 0.001	NS

PBMCs: peripheral blood mononuclear cells; CSF: cerebrospinal fluid; PPMS: primary progressive MS; RRMS: relapsing-remitting MS; SPMS: secondary progressive MS; P: positive; NS: not significant.

**Table 3:** Correlation of HHV-6-DNA detection in separate specimens (HHV-6^+^) with HHV-6 seroprevalence (IgG and IgM) in MS patients.

Correlation between variables among patients						
	IgG	IgM	Serum	PBMCs	CSF	Saliva
IgG						
Pearson correlation	1	0.429*	0.283	0.233	0.092	−0.075
Sig. (2-tailed)		0.041	0.066	0.104	0.787	0.874
IgM						
Pearson correlation	0.429*	1	0.407*	−0.193	0.111	−0.116
Sig. (2-tailed)	0.041		0.035	0.355	0.745	0.805
Serum						
Pearson correlation	0.283	0.407*	1	0.363*	0.607*	0.218
Sig. (2-tailed)	0.066	0.035		0.014	0.048	0.604
PBMCs						
Pearson correlation	0.233	−0.193	0.363*	1	0.001	0.291
Sig. (2-tailed)	0.104	0.355	0.014		0.998	0.447
CSF						
Pearson correlation	0.092	0.111	0.607*	0.001	1	·^a^
Sig. (2-tailed)	0.787	0.745	0.048	0.998		·^a^
Saliva						
Pearson correlation	−0.075	−0.116	0.218	0.291	·^a^	1
Sig. (2-tailed)	0.874	0.805	0.604	0.447	·^a^	

*Correlation is significant at the 0.05 level (2-tailed).

^a^Cannot be computed because at least one of the variables is constant.

**Table 4:** Correlation of HHV-6-DNA detection in separate specimens (HHV-6^+^) with HHV-6 seroprevalence (IgG and IgM) in healthy controls.

Correlation between variables among controls					
	IgG	IgM	Serum	PBMCs	Saliva
IgG					
Pearson correlation	1	0.665	0.791**	0.395*	−1.000*
Sig. (2-tailed)		0.072	0.000	0.012	0.010
IgM					
Pearson correlation	0.665	1	0.692	−0.012	−0.188
Sig. (2-tailed)	0.072		0.057	0.977	0.880
Serum					
Pearson correlation	0.791**	0.692	1	−0.093	0.560
Sig. (2-tailed)	0.000	0.057		0.723	0.622
PBMCs					
Pearson correlation	0.395*	−0.012	−0.093	1	0.474
Sig. (2-tailed)	0.012	0.977	0.723		0.686
Saliva					
Pearson correlation	−1.000*	−0.188	0.560	0.474	1
Sig. (2-tailed)	0.010	0.880	0.622	0.686	

**Correlation is significant at the 0.01 level (2-tailed).

*Correlation is significant at the 0.05 level (2-tailed).
